# Cross-Modal Synergy Representation of EMG and Joint Angular Acceleration During Gait in Parkinson’s Disease Using NMF and Multimodal Matrix Factorization

**DOI:** 10.3390/s26061853

**Published:** 2026-03-15

**Authors:** Jiarong Wu, Qiuxia Zhang, Wanli Zang

**Affiliations:** School of Physical Education, Soochow University, Suzhou 215021, China; 20244206013@stu.suda.edu.cn

**Keywords:** Parkinson’s disease, gait, electromyography, muscle synergies, NMF, multimodal matrix factorization, angular acceleration

## Abstract

The aims of this research were to characterize neuromuscular control features within the gait cycle in Parkinson’s disease (PD) from the perspectives of muscle synergies and cross-modal coupling and to propose a joint representation of the relationship between muscle activation patterns and kinematic dynamic outputs. PD participants (*n* = 19) were included. Lower-limb surface electromyography (EMG) and kinematic dynamic channels, including pelvic/hip, knee, and ankle angular acceleration, were collected during level-ground natural walking. EMG signals were first decomposed using non-negative matrix factorization (NMF) to extract muscle synergies, and the number of synergies was evaluated using reconstruction performance (R^2^). Multimodal matrix factorization (MMF) was then applied to jointly decompose the EMG and angular-acceleration channels, yielding a cross-modal synergy representation comprising a shared temporal structure (H) and modality-specific weight structures (W): non-negativity was imposed on EMG weights, whereas kinematic weights were allowed to take positive and negative values to encode directional contributions. Under the current task and muscle set, NMF achieved high EMG reconstruction performance with four synergies (R^2^ = 0.882). The synergy weights showed an ankle-dominant pattern: tibialis anterior (TA) consistently carried high weights across multiple synergies, while lateral gastrocnemius (LG) and soleus (SOL) contributed prominently to another synergy. The synergy activation profiles exhibited phase-dependent fluctuations with multiple rises and falls across the gait cycle, suggesting that synergy output was primarily characterized by continuous modulation rather than single-peak recruitment. MMF further identified eight cross-modal synergies, simultaneously capturing the shared contributions of key muscle groups (e.g., RF, TA, and SOL) and pelvic/hip and knee/ankle angular-acceleration channels within the same decomposition framework and summarizing their descriptive co-variation through the shared temporal structure (H). Overall, A low-dimensional synergy analysis combining EMG-only NMF with cross-modal MMF enables simultaneous characterization of cohort-level modular organization of muscle activity during gait and its descriptive association with pelvis-to-lower-limb dynamic output. This joint framework provides a methodological basis for quantitatively describing gait-related modular organization and temporal modulation patterns in this PD cohort under natural level-ground walking and lays the groundwork for subsequent testing of associations between synergy features and gait phenotypes, clinical severity, and rehabilitation responses.

## Simple Summary

Parkinson’s disease often affects walking, increasing the risk of falls and reducing independence and quality of life. To improve understanding of how walking is controlled in Parkinson’s disease, we studied 19 people while they walked at a natural pace. We recorded electrical activity from key leg muscles using skin sensors and measured how quickly the pelvis, hip, knee, and ankle joints sped up or slowed down during the walking cycle. We then used a computer method that identifies a small set of repeating patterns in complex data and another method that links muscle patterns and movement patterns using the same timing structure. We found that four main muscle patterns could describe most of the muscle activity, and these patterns were strongly influenced by ankle-related muscles. When muscle signals and joint speed-up and slow-down signals were analyzed together, eight combined patterns were identified, showing how changes in muscle activity co-varied with joint angular-acceleration dynamics across the whole body, from the pelvis to the ankle. This framework offers a clearer, measurable way to describe gait-related neuromuscular and kinematic patterns in this Parkinson’s disease cohort and may help future work validate these patterns against matched healthy references and clinically meaningful tasks.

## 1. Introduction

Parkinson’s disease (PD) is a progressive neurodegenerative disorder characterized by degeneration of the nigrostriatal dopaminergic pathway, with cardinal clinical features including bradykinesia, rigidity, and resting tremor [1,2]. With the accelerating global ageing trend, the burden of PD in terms of prevalence and disability is expected to continue rising over the coming decades [3,4]. Although treatments such as levodopa and deep brain stimulation confer clear benefits for some core motor symptoms, gait- and balance-related impairments often represent more persistent “residual problems” that remain common and enduring, making rehabilitation training and exercise interventions key components of long-term management [5,6,7].

Within the spectrum of motor impairment in PD, gait dysfunction and deficits in postural control are directly linked to fall risk, activity limitation, and reduced quality of life [8,9]. Previous systematic reviews and follow-up studies have shown that reduced gait stability in PD is strongly associated with falls; notably, increased fluctuations in gait rhythm and step-to-step timing (e.g., elevated step-time variability) have been repeatedly reported and can predict fall propensity [10,11]. From an output-phenotype perspective, PD gait commonly manifests as reduced walking speed, shortened step length, and abnormal modulation across the gait cycle. At the biomechanical level, diminished propulsive-related kinetic output during terminal stance and pre-swing has also been observed (e.g., insufficient ankle plantarflexion moment and power generation), along with kinematic abnormalities related to proximal segmental stability (e.g., increased mediolateral motion of the head–pelvis system) [10,12,13,14]. Collectively, these lines of evidence suggest that relying solely on spatiotemporal parameters or single-joint metrics can describe “how one walks” but is insufficient to explain “how control is organized to produce it”.

To better characterize gait control features in PD and their neuromuscular organization, research attention has progressively shifted from “output metrics” toward the “organization of neuromuscular control”. The muscle synergy framework posits that the central nervous system may generate complex movements through a limited number of modular muscle-group combinations and their temporal modulation, thereby enabling dimensionality reduction in control [15]. Methodologically, non-negative matrix factorization (NMF) has been widely used to extract synergy weights and activation profiles from multi-muscle electromyography (EMG) because of its interpretability and robustness [16,17,18]. In periodic tasks such as walking, synergy analysis can disentangle “which muscle groups are co-recruited as a functional unit” from “how these units are temporally organized across gait phases”, providing a structured interface for linking neural control with movement output [19,20].

Existing studies on gait synergies in PD generally suggest that, compared with healthy controls, PD may exhibit reduced control complexity (e.g., fewer synergies required to reach the same explained-variance threshold or apparent module merging) and more prominent abnormalities in temporal modulation (i.e., altered activation profiles), whereas systematic reorganization of synergy weight vectors is not consistently the primary source of between-group differences [21,22,23]. For example, Rodriguez et al. reported that, after comparing synergy structures during walking between PD and healthy older adults, group differences were more concentrated in synergy activation profiles and temporal organization rather than in large-scale rearrangement of weight vectors [21]. Subsequent reviews and empirical studies have likewise emphasized that synergy abnormalities in PD often manifest as reduced capacity for phase-specific modulation, constrained module independence, and a tendency toward “merging” of synergies related to stability and postural control [22]. However, a key limitation is that much of the literature remains at the level of EMG-only synergies, making it difficult to address which changes in kinematic dynamic outputs correspond to temporal alterations within a given muscle module, thereby limiting the value of synergy metrics for interpreting gait phenotypes, tracking intervention effects, and enabling wearable translation.

To address this gap, a “kinematics–EMG cross-modal synergy/coupling” approach has recently emerged: kinematic variables and EMG are embedded within a single decomposition framework, such that “muscle-module weights” and “kinematic-channel contributions” are estimated simultaneously under a shared low-dimensional temporal structure, thereby yielding a more direct control–output correspondence [24,25,26]. In this context, multimodal or mixed matrix factorization (MMF) allows for non-negativity constraints to be imposed on EMG weights while preserving signed (positive/negative) directionality in kinematic weights, enabling directional contributions to dynamic outputs, such as joint/segment acceleration and deceleration, to be represented while maintaining physiological interpretability [25,27]. Moreover, dynamic variables, such as angular acceleration, are highly isomorphic to inertial measurement unit (IMU) signals, offering a practical pathway for developing interpretable digital gait biomarkers in real-world settings [26,28]. Therefore, jointly applying EMG-only synergies and cross-modal synergies may integrate “modular organization” with “dynamic output” on a common scale, providing a more traceable, verifiable, and translatable chain of evidence for interpreting gait-related control features in PD and supporting future validation against healthy references.

Against this background, we adopted “muscle synergies plus cross-modal coupling” as the central framework of this study. During level-ground natural walking, we simultaneously collected lower-limb surface EMG and kinematic dynamic channels, including pelvic/hip, knee, and ankle angular acceleration, in participants with PD and implemented a two-step complementary strategy. First, NMF was used to extract EMG-only muscle synergies to characterize module composition and temporal recruitment. Second, MMF was applied to jointly decompose EMG and angular-acceleration channels, constructing a cross-modal synergy representation comprising a shared temporal structure (H) and modality-specific weight structures (W), thereby quantitatively summarizing the shared temporal structure and descriptive co-variation between “muscle activation patterns” and “kinematic dynamic outputs”. We aimed to provide a set of operational quantitative metrics to more finely describe gait-related modular organization and temporal modulation patterns in this PD cohort under natural level-ground walking and to establish a methodological basis for subsequent association and validation analyses (e.g., against matched healthy references and clinically meaningful task conditions).

## 2. Materials and Methods

### 2.1. Participants and Ethics

Data for this study were obtained from a dataset accessed via Figshare [29]. Participants with PD were recruited between September 2024 and March 2025 at the social promotion association “La Tartaruga” in Pisa, Italy. A total of 34 individuals with PD completed a baseline assessment. After the baseline, 10 participants withdrew for organizational or personal reasons (5 in the NW group and 5 in the APA group). Participants with missing EMG data were also excluded. Ultimately, 19 participants were included in the analysis (16 men/3 women; age, 76.21 ± 6.11 years; body mass, 74.47 ± 12.37 kg; height, 1.68 ± 0.08 m).

Inclusion criteria were: (1) a diagnosis of PD with Hoehn and Yahr stage 1–3; (2) ability to walk independently for >200 m during the 6 min walk test (6MWT); (3) an Addenbrooke’s Cognitive Examination-III (ACE-III) score ≥ 71; (4) stable antiparkinsonian medication dosage for at least 4 weeks before enrollment; (5) provision of a medical certificate confirming suitability for participation in non-competitive physical activity; and (6) all assessments performed in the medication ON state to reduce motor fluctuations. Exclusion criteria were withdrawal of informed consent or inability to continue the study procedures for organizational/personal reasons.

All participants provided written informed consent in accordance with the Italian legal framework. The study complied with the Declaration of Helsinki and was approved by the Bioethics Committee of the University of Pisa (approval number: 58/2024) [29].

### 2.2. Gait Task and Acquisition Procedures

Participants walked barefoot at a self-selected natural speed along a straight, level walkway of approximately 6 m. To avoid conscious adjustments in gait patterns, participants were not instructed to step onto a force platform (two AMTI OR6-7-1000 force plates, AMTI, Watertown, MA, USA; sampling frequency, 1000 Hz). At least five walking trials were recorded for each participant; three trials were selected for subsequent analyses based on signal completeness, excluding trials with evident missing data.

### 2.3. Experimental Equipment and Data Acquisition

All experiments were conducted at the Rehabilitation and Sports Medicine Center of the University of Pisa, Italy. Kinematic data were captured using eight Vicon Vero infrared cameras (Vicon Motion Systems Ltd., Oxford, UK) at a sampling frequency of 100 Hz and were recorded and processed using Vicon Nexus 2.16.1 (Vicon Motion Systems Ltd., Oxford, UK). A total of 55 reflective skin markers (14 mm diameter; 2 mm base) were placed according to the full-body PyCGM2.5 model (Supplementary Figure S1) [29] to obtain kinematic data during natural walking. Kinematic signals were processed using a zero-phase low-pass Butterworth filter (cut-off frequency, 6 Hz) to reduce noise while retaining physiologically relevant components. Based on the zero-phase low-pass filtered joint-angle time series, numerical differentiation was performed. Joint angular velocity (θ′) was first computed, and angular acceleration (θ″) was then obtained by differentiating the velocity signal. Importantly, differentiation was applied to the filtered signals to mitigate noise amplification inherent to numerical derivatives. Surface electromyography (EMG) was acquired using a 10-channel EMG system (Cometa srl, Bareggio, Italy) at a sampling frequency of 2000 Hz. Raw EMG signals were first filtered with a fourth-order Butterworth band-pass filter (20–500 Hz) to attenuate motion artifacts and high-frequency noise, and a 50 Hz notch filter was applied to suppress power-line interference. All filtering procedures were implemented using zero-phase, bidirectional filtering to avoid phase distortion. After band-pass and notch filtering, EMG signals were full-wave rectified (absolute value transformation) to obtain a non-negative envelope signal. Subsequently, each EMG channel was amplitude-normalized within a single task cycle by its maximum value.

### 2.4. Data Processing

#### 2.4.1. Time-Window Selection and Time Normalization

Although a force-plate system was available in the experimental setup, participants were not instructed to deliberately step onto the force plates to avoid conscious adjustments that could alter natural gait. As a result, it could not be ensured that each step fully landed on the force plates, and step-by-step gait-cycle segmentation based on heel-strike events or force-plate signals was not adopted. Instead, a fixed time-window strategy was used to extract a stable walking segment for analysis. The first 0.1 s of data after acquisition onset were discarded to minimize the influence of gait initiation and initial transient instability. Subsequently, a continuous 4 s segment of steady-state walking was selected as the analysis window. Within the typical range of self-selected cadence during natural walking, this window generally contains multiple consecutive gait cycles, thereby capturing steady gait behavior and improving the stability of co-variance-structure estimation for synergy analyses. EMG and kinematic data extracted from the same window were strictly time-aligned, resampled to 101 equally spaced time points, and linearly mapped to a normalized time scale of 0–100%. Notably, in the present study, “0–100%” represents the normalized time axis of the selected steady walking window rather than a single complete gait cycle.

#### 2.4.2. NMF

To extract EMG-only muscle synergies from EMG envelopes, NMF was applied to decompose a non-negative data matrix [30]. Let *X* be an *m* × *n* non-negative data matrix, where each column is a sample vector; NMF approximates *X* as the following:

X=WH+E

where *E* is the error term, and the dimensions of *W* and *H* are *m* × *r* and *r* × *n*, respectively. When *E* ≈ 0, the above expression can be simplified as follows:

X=WH

The decomposition of *X* into *W* and *H* is obtained by minimizing an error function between the original data matrix and its factorized approximation. Different cost functions can be used for NMF optimization. Lee and Seung proposed two cost functions and provided corresponding update rules for *W* and *H* [17,31]. The most commonly used and simplest cost function is the Euclidean distance:

*J* = ||*X* − *WH*||

This cost function is minimized under the constraints W, H≥0. NMF was performed separately on each participant’s data to obtain the corresponding spatial weight matrix (W) and temporal activation matrix (H), thereby preserving individual neuromuscular control characteristics and avoiding cross-participant co-variance mixing.

#### 2.4.3. MMF

MMF is an extension of NMF that decomposes a data matrix containing both non-negative signals and unconstrained signals [26]. Because the factorization rank (i.e., the number of extracted synergies, N) is a free parameter, MMF is typically called repeatedly within a loop, with N increasing from 1 up to the number of rows in the data matrix, *D*. For each factorization rank, both the synergy matrix and the coefficient matrix are randomly initialized and iteratively updated using a gradient descent algorithm. Let *W* denote the synergies and *C* denote the temporal coefficients. The update rules derived from the gradient of a cost function incorporating the reconstruction error ||D−WC|| and a norm term on W are as follows:

$$W=W+2\mu \left[\left(D-WC\right)C^{T}-\lambda W\right]$$

$$C=C+2\mu \left[W^{T\left(D-WC\right)}\right]$$

where the learning rate is defined as *μ* = *μ*_ratio_/‖*D*‖, and the regularization weight is defined as *λ* = *λ*_ratio_/(N⋅sample), consistent with the settings in [25]. The learning rate and regularization weight were defined in a data-adaptive manner as *μ* = *μ*_ratio/‖*D*‖_F and *λ* = *λ*_ratio/(N · S), where *D* denotes the normalized mixed data matrix, ‖*D*‖_F denotes its Frobenius norm, N the factorization rank, and S the number of samples. The proportional constants were set to *μ*_ratio = 0.1 and *λ*_ratio = 50. Optimization was performed using gradient descent with random initialization of *W* and *C*. A minimum of 100 iterations was enforced, and the procedure terminated when the improvement in reconstruction quality over 10 consecutive iterations was below 10^−6^, with a maximum of 10,000 iterations. For each N, the factorization was repeated 10 times, and the solution with the highest reconstruction quality was retained to reduce the risk of being trapped in local minima [26]. Non-negativity is enforced by constraining selected rows of *W*: at each optimization iteration, certain rows (e.g., those containing EMG signals) are restricted to be non-negative or zero. Iterative updates stop when the convergence criterion is met. The default convergence criterion is that, over 10 consecutive iterations, the improvement in reconstruction quality is smaller than the error convergence threshold. Reconstruction quality, R^2^, is defined as follows:

$$R^{2}=1-\frac{SSE}{SST}=1-\frac{tr\left[\left(D-WC\right){\left(D-WC\right)}^{T}\right]}{tr\left[\left(D-\bar{D}\right){\left(D-\bar{D}\right)}^{T}\right]}$$

where tr (·) denotes the matrix trace, and D denotes the mean matrix of the data matrix, D (centered according to the corresponding definition). To reduce the likelihood of being trapped in local minima, for each N, the optimization was repeated 10 times, and the solution with the highest reconstruction quality was selected as the final result. To ensure numerical balance between modalities in mixed matrix factorization, amplitude normalization was applied to all channels prior to decomposition. After time normalization, kinematic signals were linearly scaled to the range of [−1, 1]. Following full-wave rectification and max-value normalization, EMG signals were further rescaled to [0, 2] so that their amplitude span was comparable to that of the kinematic signals. This procedure was intended to prevent signals with different units and scales from contributing disproportionately to the reconstruction error [26].

## 3. Results

### 3.1. Joint-Angle and Angular-Acceleration Characteristics

**Figure 1:**
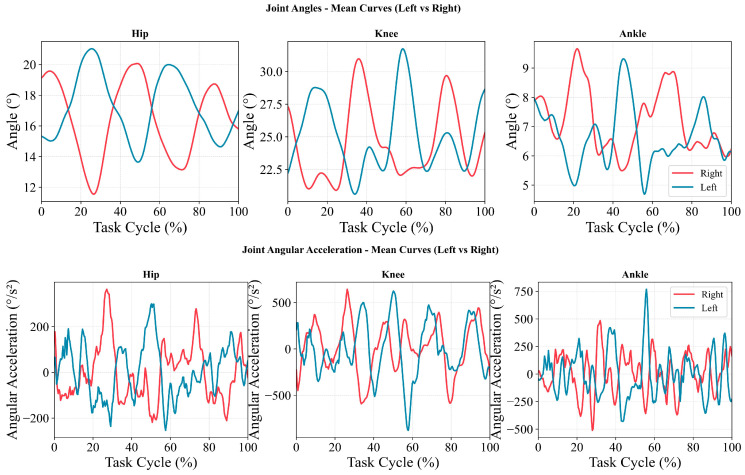
Mean hip, knee, and ankle joint angles (**top row**) and joint angular accelerations (**bottom row**) of the left and right legs across the task cycle.

Figure 1 summarizes the across-participant mean curves for left and right hip, knee, and ankle joint angles (upper panels) and angular acceleration (lower panels) over the normalized gait cycle (0–100%) in 19 participants. Overall, joint angles across the three joints exhibited stable periodic patterns, with visible phase offsets between left and right curves in certain phases. Hip joint angles varied relatively smoothly. Knee joint angles showed larger fluctuations with more pronounced peak-to-trough transitions. Ankle joint angles exhibited a multi-peak pattern, with left–right differences in the timing of local peaks and troughs.

Angular-acceleration curves alternated between positive and negative values and displayed intermittent peaks and troughs across the gait cycle, indicating phase-dependent periods of acceleration and deceleration in joint rotational dynamics. Compared with the hip, knee and ankle angular-acceleration peaks were larger in magnitude (hip: approximately ±300–350 °/s^2^; knee: approximately ±600–800 °/s^2^; ankle: approximately ±700–800 °/s^2^). Left–right differences were mainly reflected in the timing of peak occurrences and the distribution of positive and negative polarity.

### 3.2. NMF-Based Muscle Synergy Structure and Temporal Recruitment Characteristics

#### 3.2.1. Determination of the Number of Synergies (R^2^–Synergy Order Relationship)

The optimal number of synergies was determined using the R^2^ plateau criterion, and the selected model order converged to four synergies in all 19 participants. When the number of synergies increased from 4 to 5, the mean gain in R^2^ was <2% (below the 5% threshold), supporting the choice of four synergies. With four synergies, the subject-wise R^2^ distribution was: mean ± SD = 0.882 ± 0.044, median [IQR] = 0.874 [0.849–0.904], and range = 0.800–0.981 (Supplementary Figure S2). All participants achieved an R^2^ > 0.80, consistent with prior synergy studies [32,33].

#### 3.2.2. Synergy Weight Composition (W) and Dominant Muscle Contributions

**Figure 2:**
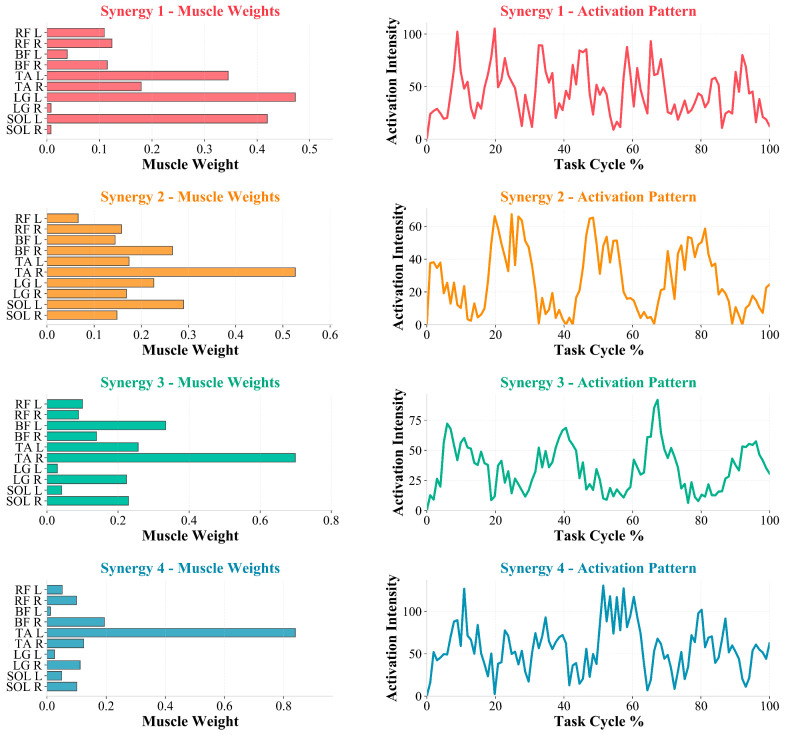
Muscle synergy weights and activation patterns (Synergies 1–4). Left: Muscle weighting coefficients showing each muscle’s contribution to the four identified synergies. Right: Temporal activation profiles of each synergy across the task cycle (0–100%).

Figure 2 (left column) presents the weight matrix (W) of the four synergies, and Table 1 summarizes representative muscles with higher weights within each synergy (mean ± SD). Overall, synergy weights were dominated by ankle-centered muscle groups, with tibialis anterior (TA), lateral gastrocnemius (LG), and soleus (SOL) carrying relatively high weights across multiple synergies.

By synergy, Syn1 was primarily characterized by contributions from left plantarflexion-related muscles, mainly LG (L) (0.472 ± 0.327) and SOL (L) (0.419 ± 0.213), together with TA (L) (0.345 ± 0.454). Syn2 was dominated by the right dorsiflexor TA (R) (0.526 ± 0.237), followed by SOL (L) (0.289 ± 0.281) and biceps femoris (BF) (R) (0.266 ± 0.253). Syn3 was likewise dominated by TA (R) (0.699 ± 0.126), accompanied by BF (L) (0.334 ± 0.236) and TA (L) (0.257 ± 0.252). Syn4 showed a left dorsiflexion-dominant pattern, with TA (L) having the highest weight (0.839 ± 0.216), whereas other muscles contributed relatively less (Table 1).

#### 3.2.3. Temporal Activation Patterns of Synergies

Figure 2 (right column) shows the activation matrix of each synergy over the normalized gait cycle (0–100%). Overall, all four synergies exhibited phase-dependent modulation across the gait cycle and multiple local peaks, suggesting that muscle modules underwent intermittent recruitment and sustained modulation within the cycle. Specifically, the right TA-dominant synergies (Syn2–Syn3) showed relatively more concentrated intervals of increased activation, whereas the left TA-dominant Syn4 and the left plantarflexion-related Syn1 more often displayed multi-peak activation patterns. These differences suggest phase-dependent recruitment patterns across distinct muscle modules over the gait cycle.

### 3.3. MMF-Based Cross-Modal Synergy Results

#### 3.3.1. Determination of the Number of Synergies

Based on the VAF ≥ 0.80 criterion, together with the diminishing marginal-gain rule [32], eight cross-modal synergies were ultimately retained for subsequent analyses (Supplementary Figure S3). With eight synergies, the subject-wise reconstruction performance showed: mean ± SD = 0.855 ± 0.017, median [IQR] = 0.854 [0.844–0.866], and range = 0.814–0.899; all participants met the R^2^ > 0.80 criterion, and between-subject variability was minimal (SD = 0.017), indicating good cross-participant reconstruction stability of the MMF model.

#### 3.3.2. Weight Matrix (W) and Dominant Channel Contributions (EMG and Kinematics)

**Figure 3:**
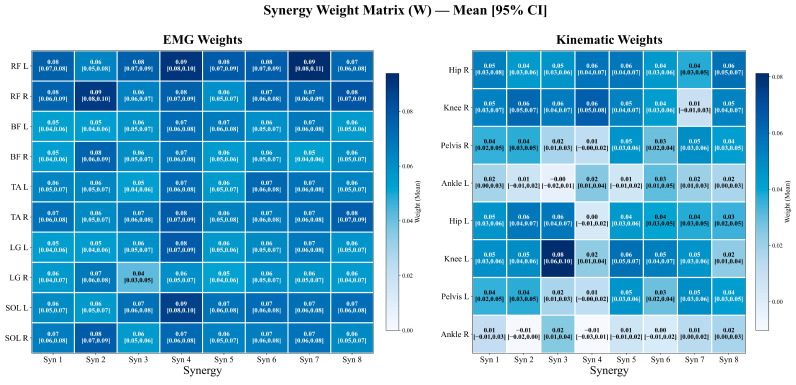
Heatmap of the synergy weight matrix (W) for EMG (left) and kinematic (right) channels across eight synergies. Cell values represent group mean weights with 95% confidence intervals. Color intensity indicates weight magnitude. EMG weights are non-negative (NMF constraint); kinematic weights are unconstrained, allowing for negative values.

Figure 3 shows a heatmap of the weight matrix (W) for the eight cross-modal synergies (mean and 95% confidence intervals), with EMG-channel weights on the left and kinematic-channel (joint angular-acceleration) weights on the right.

For the EMG weights, muscle groups such as RF and SOL exhibited relatively higher weights across multiple synergies. Overall, Synergy 4 showed a more prominent EMG weight distribution (e.g., RF L: 0.09 [0.08, 0.10], SOL L: 0.09 [0.08, 0.10], LG L: 0.08 [0.07, 0.09], and TA R: 0.08 [0.07, 0.09]). In addition, RF L in Synergy 7 reaches a comparatively high value among the EMG entries in this figure (0.09 [0.08, 0.11]). Across EMG channels, weights were generally distributed within approximately 0.04–0.10 and were consistently positive (consistent with the non-negativity constraint imposed on EMG weights in MMF), indicating positive-direction contributions of the muscle channels to the cross-modal synergies.

For the kinematic weights, Hip and Knee (and, in some cases, Pelvis) angular-acceleration channels showed relatively higher weights in most synergies. For example, Knee L in Synergy 3 had the highest kinematic weight (0.08 [0.06, 0.10]). In contrast, Ankle angular-acceleration channels tended to have lower weights overall, and their 95% confidence intervals included zero in multiple synergies (e.g., Ankle R in Synergy 2: −0.01 [−0.02, 0.00]; Synergy 4: −0.01 [−0.03, 0.01]), suggesting smaller contribution magnitudes and less consistent directionality across participants for ankle angular acceleration in these synergies. Similarly, Pelvis and Hip channels also showed 95% confidence intervals that included zero in specific synergies (e.g., in Synergy 4, Pelvis R: 0.01 [−0.00, 0.02], Hip L: 0.00 [−0.01, 0.02]), indicating greater between-participant variability in their contributions under certain synergy patterns.

#### 3.3.3. Temporal Patterns of Activation Coefficients (H) over the Task Cycle

**Figure 4:**
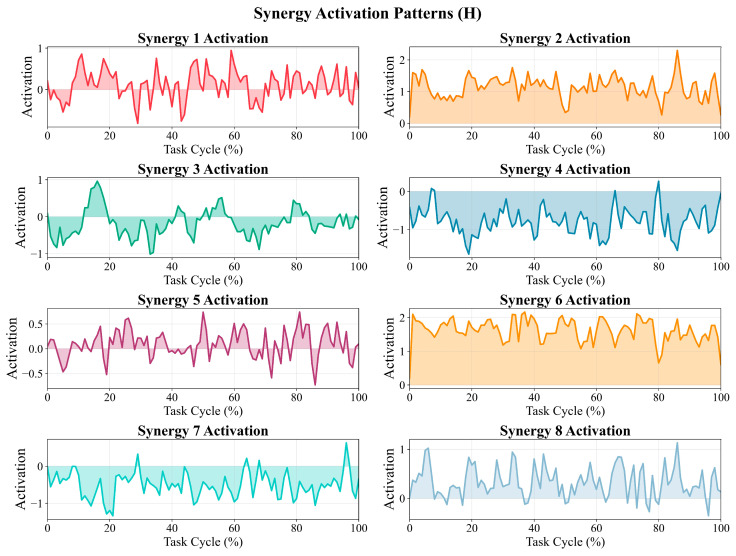
Synergy activation coefficient matrix (H) showing temporal patterns across the task cycle.

Figure 4 illustrates the temporal coefficients (H) of the eight cross-modal synergies over the normalized gait cycle (0–100%). Overall, all synergies exhibited multiple rises and falls with local peaks and troughs throughout the cycle, indicating sustained temporal modulation of cross-modal synergy contributions rather than a strictly single-peak, pulse-like activation pattern. In terms of sign and amplitude characteristics, Syn2 and Syn6 remained strictly positive throughout the cycle (Syn2: ranging from 0.21 to 2.30; Syn6: ranging from 0.21 to 2.17), maintaining relatively sustained positive levels with superimposed local peaks. Notably, Syn2 demonstrated a more pronounced peak toward the late phase of the cycle (approximately 80–90%). In contrast, Syn4 and Syn7 were predominantly negative (Syn4: ranging from −1.64 to 0.27; Syn7: ranging from −1.34 to 0.63), maintaining a negative baseline with intermittent fluctuations. Syn3 exhibited a marked positive local peak early in the cycle (approximately 15–20%), followed by predominantly negative values with repeated oscillatory fluctuations (ranging from −1.01 to 0.96). Syn1 and Syn5 showed alternating positive and negative fluctuations (Syn1: ranging from −0.81 to 0.95; Syn5: ranging from −0.73 to 0.74), characterized by multiple local maxima and minima across phases. Syn8 was predominantly positive with multiple local excursions (ranging from −0.36 to 1.13), including a more evident peak toward the end of the cycle. It should be noted that the absolute magnitude and sign of H depend on the specific factorization and normalization procedures applied. Therefore, H should primarily be interpreted as a relative temporal modulation pattern across the normalized cycle and considered in conjunction with the corresponding weight matrix (W) when analyzing cross-modal synergy structure.

## 4. Discussion

### 4.1. Main Findings of This Research

This study characterized neuromuscular control features in participants with PD over the normalized gait cycle from the perspectives of muscle synergies and cross-modal coupling. We first extracted muscle synergies using EMG-only NMF and then applied multimodal matrix factorization (MMF) to jointly decompose EMG and pelvic/hip, knee, and ankle angular-acceleration channels to obtain a joint, low-dimensional representation linking muscle-module patterns with kinematic dynamic outputs. The main findings were as follows. First, under the task and muscle set used in this study, four NMF synergies achieved high EMG reconstruction accuracy (R^2^ = 0.8817; Supplementary Figure S2), and the synergy weights showed prominent ankle-related contributions (TA, LG, and SOL; Figure 2 and Table 1), indicating that, in this PD cohort under natural level-ground walking, muscle groups related to ankle dorsiflexion/plantarflexion contribute substantially to the extracted modular patterns. Second, with eight cross-modal synergies retained in MMF (Supplementary Figure S3), the same decomposition framework provided the weight contributions of both EMG and kinematic channels (Figure 3) as well as their activation patterns across the gait cycle (Figure 4). Collectively, these results provide a quantitative basis for further discussion of ankle-related modular patterns in this PD cohort and the descriptive co-variation between muscle activity and pelvis-to-lower-limb acceleration–deceleration dynamics captured by the shared temporal structure. Because no healthy reference/control group was included, the following discussion focuses on cohort-level descriptive patterns and methodological implications; PD-specific abnormality and mechanistic/causal interpretations require validation in controlled studies.

### 4.2. EMG-Only Synergies (NMF): Module Composition and Temporal Recruitment

Our results indicate that, under the current gait task and muscle set, EMG-only NMF achieved relatively high reconstruction accuracy with four synergies (R^2^ = 0.8817). The synergy weights were characterized by prominent contributions from ankle-centered muscle groups: TA consistently carried relatively high weights across multiple synergies, whereas LG and SOL contributed prominently to another synergy. This pattern suggests that, within the present experimental setting, the main variance in gait EMG in this PD cohort was captured by a small number of low-dimensional synergies related to ankle dorsiflexion/plantarflexion and peri-ankle stability. Accordingly, we treat the selected synergy number as a descriptive model order for this dataset. Consistent with prior gait–synergy studies comparing PD and healthy controls, related work often reports that fewer synergies are required in PD to reach a fixed explained-variance threshold, while differences in spatial weights are relatively limited; between-group differences are more prominently reflected in changes in the timing and amplitude of synergy activation profiles, supporting an interpretive framework of “reduced control complexity accompanied by altered temporal modulation” [21]. Moreover, previous studies have further interpreted fewer synergies in PD as reflecting merging of distinct biomechanical functions and reduced module independence, potentially involving constrained independent control of synergies related to body stability and dynamic postural control [34]; these interpretations warrant direct validation using matched healthy controls and task conditions that elicit hallmark PD gait features.

At the temporal level, activation profiles of all synergies in this study showed multiple rises and falls and local peaks and troughs over the normalized gait cycle, rather than single-peak pulse-like recruitment with clear phase boundaries. This pattern suggests repeated modulation of the same synergy within the cycle, with more dispersed activation and more fragmented peak occurrence, indicating that phase-dependent synergy output may be less clearly expressed as a “concentrated–switching” temporal organization in this PD cohort under the current task. This observation aligns with conclusions from prior NMF-based gait–synergy research. Rodriguez et al. reported that, when comparing PD with healthy older controls under the same explained-variance threshold, group differences were mainly reflected in alterations of synergy activation profiles/temporal features, whereas synergy weight vectors were relatively stable, suggesting that between-group differences may be more attributable to altered temporal modulation of existing modules than to systematic reorganization of module composition [21]. In addition, surface EMG studies during level-ground walking have reported that PD (including early PD), compared with healthy controls, can exhibit reduced phase-specific modulation capacity of key muscles across the gait cycle (e.g., a reduced TA modulation index), together with a tendency toward increased coactivation/cocontraction of antagonistic lower-leg muscles. These electrophysiological findings offer contextual support for interpreting the more “dispersed/more fragmented” temporal features of synergy activation observed in this study, and these observations warrant further validation in future studies [33,34].

Regarding the synergy weight structure, we observed laterality-weighted patterns in some synergies. For example, the dominant contributions in Syn2 and Syn3 both came from TA (R). Syn4 was dominated by TA (L), whereas Syn1 showed relatively higher contributions from LG (L) and SOL (L) (Table 1). This laterality difference is potentially compatible with the common clinical phenotype of unilateral onset and persistent asymmetry of symptoms in PD; clinically, symptom laterality can typically be defined using the left/right subitems of the Movement Disorder Society-sponsored revision of the Unified Parkinson’s Disease Rating Scale (MDS-UPDRS)/Unified Parkinson’s Disease Rating Scale, Part III (UPDRS-III) [35]. However, individual-level MDS-UPDRS/UPDRS-III laterality subscores were not available in the present dataset, and thus the correspondence between synergy laterality and clinical laterality cannot be directly evaluated here. Meanwhile, gait asymmetry at the level of spatiotemporal metrics has also been repeatedly reported in PD and may relate to interhemispheric sensorimotor pathway alterations suggested in prior work. Importantly, however, gait asymmetry does not necessarily map one to one onto clinical symptom laterality, and concordance may vary at the individual level [36]. Accordingly, we consider the laterality-weighted synergy patterns observed here as a phenomenon-level finding that warrants attention, and further validation of its consistency and interpretive boundary will require clinical laterality stratification and/or individual-level analyses [37].

### 4.3. MMF-Based Cross-Modal Synergies (EMG–Kinematics Coupling)

In this study, multimodal matrix factorization (MMF) was used to jointly decompose gait EMG envelopes and pelvic/hip, knee, and ankle angular-acceleration channels, thereby representing muscle activity and dynamic output within the same low-dimensional shared temporal pattern. Compared with EMG-only NMF, MMF introduces a shared temporal component (H) and estimates modality-specific weights (W), providing a joint representation of muscle channels and kinematic channels within the same synergy component: non-negativity constraints are imposed on EMG weights to preserve physiological interpretability, whereas kinematic weights are allowed to take positive and negative values to represent directional (positive/negative) contributions to angular acceleration. Accordingly, each cross-modal synergy contains both a “weighted combination of muscle channels” and its corresponding “contributions from angular-acceleration channels” within the shared temporal structure (H), as summarized in Figure 3 using mean weights with 95% confidence intervals.

From the weight structure (W; Figure 3), the extracted synergies show recurring “muscle–kinematic channel” combinatorial representations, rather than being dominated by a single muscle group or a single joint channel. Across multiple synergies, relatively higher-weight EMG channels repeatedly involve muscle groups such as RF, TA, and SOL, while on the kinematic side, angular-acceleration channels from Pelvis/Hip as well as Knee (and, to a lesser extent, Ankle) frequently enter the main contribution sequence. This pattern suggests that, under the shared temporal structure of MMF, muscle-recruitment patterns and dynamic characteristics of both proximal (pelvis/hip) and distal (knee/ankle) segments can be jointly summarized at the synergy level. Here, we interpret these cross-modal patterns as descriptive co-variation within this PD cohort rather than PD-specific or mechanistic coupling. Notably, for some kinematic channels, the 95% confidence intervals of the weights in Figure 3 include zero, suggesting that the direction and/or magnitude of their contributions may be less consistent across participants in some synergy components. When viewed alongside existing biomechanical evidence on PD gait, review-level summaries based on kinematic–kinetic measurements suggest that PD gait often shows a reduced ankle extension moment and power generation during terminal stance/pre-swing, together with kinetic changes, such as reduced anteroposterior ground reaction forces around the propulsive phase, corresponding to a phenotype of constrained push-off/propulsive output [14]. PD gait has also been repeatedly reported to exhibit fluctuations related to rhythm and stability (e.g., increased gait-cycle time variability), with altered spatiotemporal parameters associated with fall risk; in PD populations at higher fall risk, features such as greater mediolateral motion of the head and pelvis have also been observed [10,38]. In this context, the repeated co-occurrence in MMF of “key lower-limb muscles (RF/TA/SOL)—pelvis/hip and knee/ankle angular-acceleration channels” can be viewed as a cross-modal representation that juxtaposes muscle recruitment modules and output-level dynamic features within a shared low-dimensional structure and may serve as a candidate feature interface for subsequent phenotype association analyses (e.g., correspondence with gait stability, propulsion-related indices, or clinical severity).

In the temporal structure (H; Figure 4), the temporal coefficients (H) of the eight cross-modal synergies exhibit varying degrees of phase dependence and multiple rises and falls across the 0–100% normalized task cycle, suggesting that each synergy’s contribution strength is continuously modulated over the cycle instead of showing a single sustained plateau confined to one phase. This temporal characteristic is comparable to reports from previous PD gait “modular control/synergy” studies. Rodriguez et al. noted in comparisons between PD and healthy older controls that the primary differences in PD were more prominently reflected in changes in activation profiles, whereas muscle weighting vectors were relatively more stable [21]. Mileti et al. similarly summarized that between-group differences in gait-related modular control may be more evident in altered activation timing/profiles of modules than in systematic rearrangement of weight vectors [22]. Building on this, by incorporating joint/pelvic angular-acceleration channels into the same decomposition framework, MMF enables the temporal organization at the synergy level to be presented alongside the time distribution of dynamic output channels, thereby providing a cross-modal complementary representation for describing the alignment/co-variation between “muscle recruitment timing” and “kinematic dynamic output” in this PD cohort, which warrants further validation in future studies under clinically meaningful task conditions.

### 4.4. Clinical Implications and Translational Value

By jointly applying EMG-only NMF and cross-modal multimodal matrix factorization (MMF), this study simultaneously characterized muscle synergies (EMG weights and activation timing) and their descriptive alignment/co-variation with pelvic-to-lower-limb joint angular-acceleration dynamic outputs within a unified low-dimensional representation, providing a quantitative and interpretable, structured description of gait-related neuromuscular–kinematic patterns in this PD cohort under natural level-ground walking. Because no healthy reference group was included, these findings are positioned as a proof-of-concept methodological demonstration rather than evidence of PD-specific abnormality. Compared with evaluations relying solely on spatiotemporal parameters or single EMG metrics, this framework provides standardized, interpretable outputs, such as the number of synergies, weight distributions, and activation profiles, to describe modular organization and temporal modulation and further supports uncertainty-aware interpretation by reporting subject-wise reconstruction performance and confidence intervals for cross-modal weights. It also uses the shared temporal structure to link muscle temporal organization with proximal (pelvis/hip) and distal (knee/ankle) dynamic channels within the same component, thereby providing a set of candidate metrics to support subsequent association analyses and future longitudinal evaluation of “synergy features–gait phenotypes/clinical severity/training responses”.

From the channel combinations identified in cross-modal synergies, the repeated co-occurrence of key lower-limb muscles (e.g., RF/TA/SOL) with pelvic/hip and knee/ankle angular-acceleration channels suggests that, within this PD cohort, the cross-modal representation may capture not only the recruitment organization of distal ankle-centered muscles but also the joint involvement of proximal segmental dynamic output at the synergy level. On this basis, future work may further investigate phase-specific organization of proximal stability control and distal propulsion-related muscle recruitment as hypothesis-generating directions, with cross-modal synergy metrics serving as candidate quantitative outcomes. For digital monitoring and intelligent-assistance translation, the low-dimensional temporal structures and cross-modal weight combinations derived from synergy decomposition can be used to build an interpretable gait feature library. However, their use for online identification or closed-loop interventions will require evaluation of stability and generalizability in larger samples and under more diverse task conditions, and further integration with measures such as ground reaction forces and net joint kinetics to establish a multi-level validation chain (synergy–output–function) before any clinical implementation is considered.

### 4.5. Study Limitations

Several limitations should be acknowledged. Most importantly, no healthy reference/control group was included, which precludes direct between-group inference and limits the interpretation of PD-specific abnormality; therefore, the present findings should be interpreted as cohort-level descriptive patterns and a proof-of-concept methodological demonstration. In addition, as a factorization-based, descriptive approach primarily used for dimensionality reduction and pattern summarization, NMF/MMF does not, by itself, establish mechanistic or causal relationships between muscle activity and kinematic outputs; controlled experiments and/or longitudinal designs are required for verification. Additionally, the sample size was relatively small (*n* = 19), and PD clinical phenotypes and severity are markedly heterogeneous. We did not stratify participants by tremor-dominant phenotype, postural instability and gait difficulty (PIGD), or freezing of gait (FOG) risk, which limits the generalizability and comparability of synergy features across subtypes. Accordingly, the synergy patterns reported here should be interpreted as cohort-level descriptive averages rather than subgroup-specific signatures. Averaging across heterogeneous participants may attenuate true subgroup differences and, in some cases, yield composite patterns that do not fully represent any single phenotype. Future studies with larger samples and balanced clinical subgroups (including standardized assessments of phenotype and symptom laterality) are needed to evaluate subgroup-specific synergy features and their clinical relevance. Moreover, all participants were assessed in the medication ON state. Because medication effects on motor performance and control strategies may alter synergy timing and cross-modal coupling characteristics, these findings should not be directly extrapolated to unmedicated or OFF-state conditions. Furthermore, the task was limited to level-ground natural straight walking and did not include more challenging contexts, such as turning, obstacle negotiation, speed changes, or dual-task walking; accordingly, the present study may not have fully captured synergy reorganization or potential changes in cross-modal patterns that could emerge under high-challenge conditions. Fourth, this was a cross-sectional study, and thus it cannot determine the plasticity or predictive value of synergy number, temporal pattern features, or cross-modal coupling patterns across disease progression and rehabilitation training. Longitudinal follow-up and intervention studies are needed to validate the stability and sensitivity of these metrics as phenotypic or treatment-response candidates.

## 5. Conclusions

This study proposed and implemented a low-dimensional synergy analysis framework that combines EMG-only NMF with EMG–kinematics MMF to characterize gait-related neuromuscular patterns in this PD cohort using a joint representation linking muscle modular patterns with kinematic dynamic outputs. Under the gait task and muscle set used in this study, four NMF synergies achieved relatively high EMG reconstruction performance (R^2^ = 0.8817), with prominent contributions from ankle-centered muscle groups; synergy activation across the gait cycle showed phase-dependent multi-peak modulation and some laterality-weighted patterns. MMF further identified eight cross-modal synergies and, within a single decomposition framework, used a shared temporal structure to capture the joint contributions of key muscle groups (e.g., RF/TA/SOL) and pelvic/hip, knee, and ankle angular-acceleration channels, summarizing their descriptive co-variation at the synergy level. Overall, this framework provides a quantitative and interpretable representational tool for descriptively characterizing modular organization and temporal modulation patterns in this dataset under natural level-ground walking and offers a methodological basis and candidate feature interface for future studies to test associations with gait phenotypes, clinical severity, and rehabilitation responses, including validation against matched healthy references and more challenging task conditions.

## Figures and Tables

**Table 1:** Dominant muscle contributions to each synergy.

Synergy	Top1 (Mean ± SD)	Top2 (Mean ± SD)	Top3 (Mean ± SD)	Top4 (Mean ± SD)
Syn1	LG L: 0.472 ± 0.327	SOL L: 0.419 ± 0.213	TA L: 0.345 ± 0.454	TA R: 0.179 ± 0.188
Syn2	TA R: 0.526 ± 0.237	SOL L: 0.289 ± 0.281	BF R: 0.266 ± 0.253	LG L: 0.226 ± 0.247
Syn3	TA R: 0.699 ± 0.126	BF L: 0.334 ± 0.236	TA L: 0.257 ± 0.252	SOL R: 0.230 ± 0.015
Syn4	TA L: 0.839 ± 0.216	BF R: 0.194 ± 0.274	TA R: 0.124 ± 0.174	LG R: 0.112 ± 0.148

## Data Availability

The data used in this study are publicly available in Figshare: Parkinson’s Disease Motion Analysis Dataset—Pre/Post Nordic Walking Training (https://doi.org/10.6084/m9.figshare.29371769). The accompanying processing script provided by the dataset creators is available in the same repository. No new data were created in this study.

## Supplementary Materials

The following supporting information can be downloaded at: https://www.mdpi.com/article/10.3390/s26061853/s1, Figure S1. Position of the markers according to the PYCGM2.5 model. Figure S2. Reconstruction accuracy (R^2^) as a function of the number of synergies (1–8). Figure S3. Relationship between the number of synergies and mean reconstruction performance (VAF/R^2^).

## Abbreviations

The following abbreviations are used in this manuscript:

PD	Parkinson’s disease
EMG	Surface electromyography
NMF	Non-negative matrix factorization
MMF	Multimodal matrix factorization
W	Weight matrix
H	Shared temporal structure
C	Temporal coefficients
R^2^	Reconstruction performance
VAF	Variance accounted for
IMU	Inertial measurement unit
6MWT	6-min walk test
ACE-III	Addenbrooke’s Cognitive Examination-III
UPDRS-III	Unified Parkinson’s Disease Rating Scale, Part III
MDS-UPDRS	Movement Disorder Society-sponsored revision of the Unified Parkinson’s Disease Rating Scale
PIGD	Postural instability and gait difficulty
FOG	Freezing of gait
RF	Rectus femoris
BF	Biceps femoris
TA	Tibialis anterior
LG	Lateral gastrocnemius
SOL	Soleus
L	Left
R	Right
