# Supplementary material for: Cross-Modal Synergy Representation of EMG and Joint Angular Acceleration During Gait in Parkinson’s Disease Using NMF and Multimodal Matrix Factorization
Source: Sensors (Basel). 2026 Mar 15;26(6):1853. doi: 10.3390/s26061853 (PMC13030764; doi:10.3390/s26061853)
Supplement: Supplementary file 1 [file sensors-26-01853-s001.zip › sensors-4154348-supplementary.pdf]

sensors

MDPI

Supplementary document

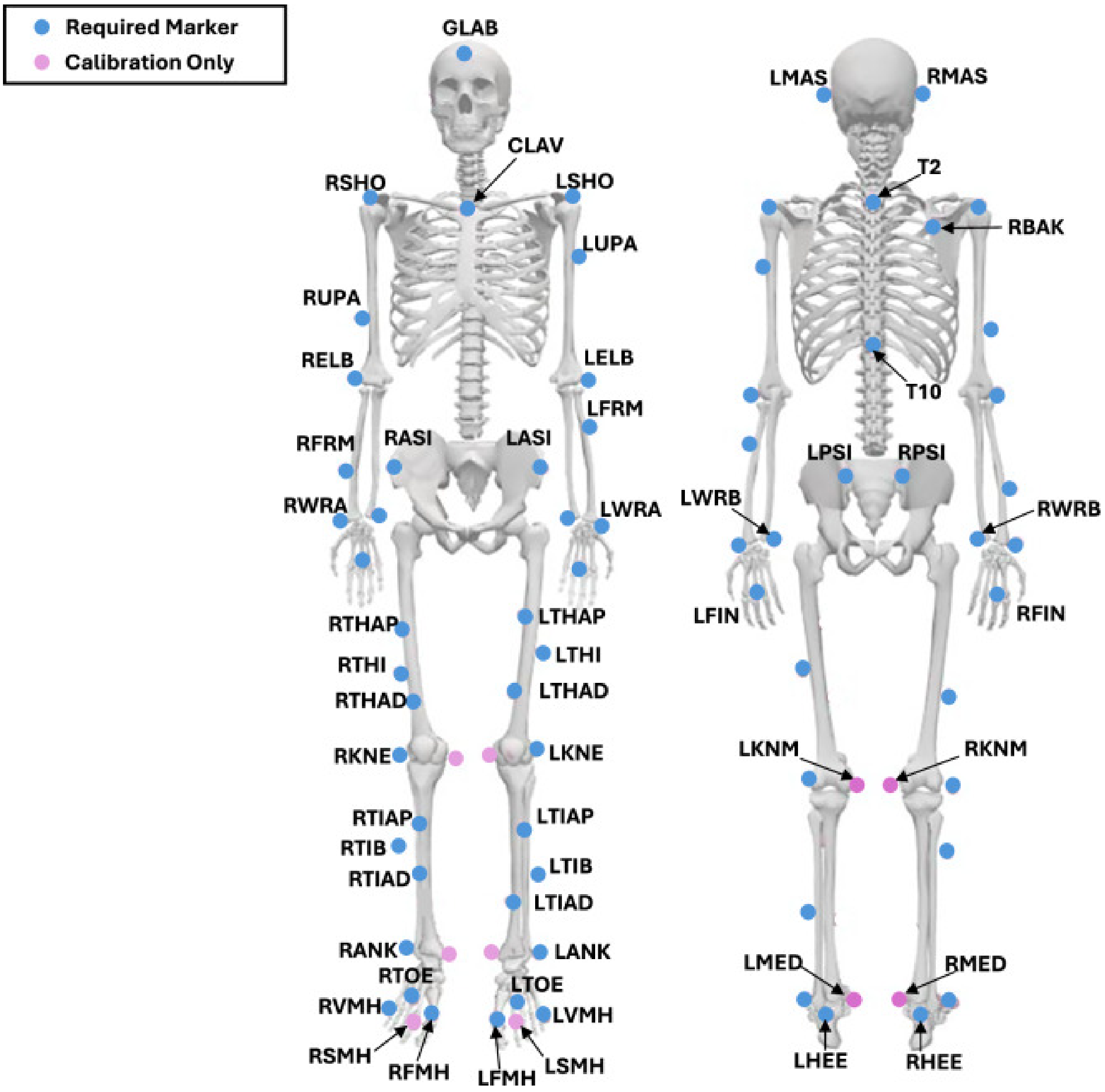

Figure S1. Position of the markers according to the PYCGM2.5 Model.

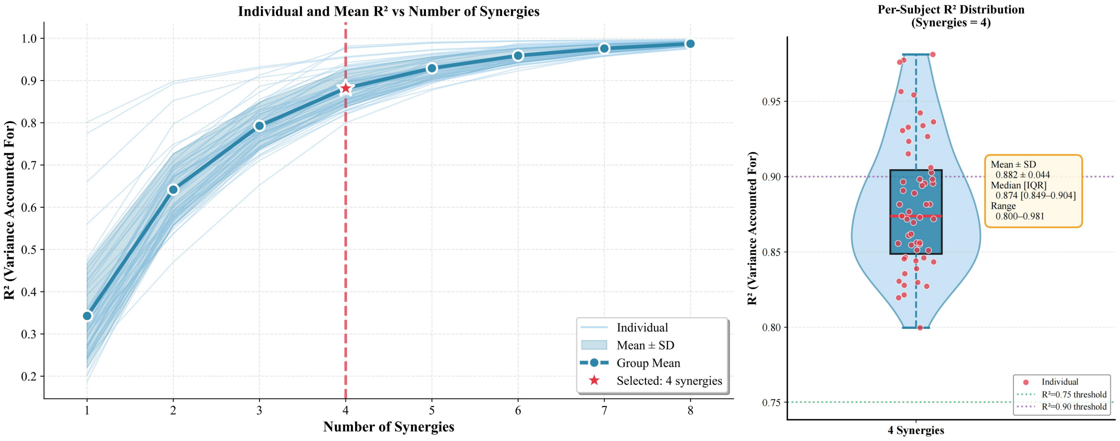

Figure S2. Reconstruction accuracy (R^2) as a function of the number of synergies (1–8).

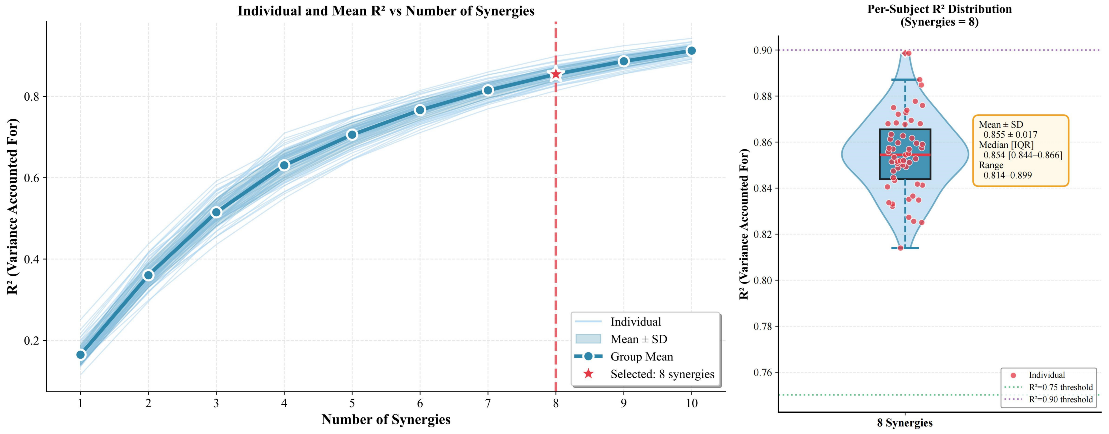

Figure S3. Relationship between the number of synergies and mean reconstruction performance (VAF/R^2).
